# Modified Intraperitoneal Chemotherapy Without Bevacizumab as a First-Line Therapy for Newly Diagnosed Advanced Epithelial Ovarian Cancer-Two Centers Experiences

**DOI:** 10.3389/fmed.2022.846352

**Published:** 2022-03-17

**Authors:** Yuanming Shen, Sangsang Tang, Junfen Xu, Xing Xie, Zhongbo Chen

**Affiliations:** ^1^School of Medicine, Women's Hospital, Zhejiang University, Hangzhou, China; ^2^Women's Reproductive Health Laboratory of Zhejiang Province, Women's Hospital, Zhejiang University, Hangzhou, China; ^3^Cancer Research Institute, Zhejiang Cancer Hospital, Hangzhou, China

**Keywords:** intraperitoneal chemotherapy, advanced epithelial ovarian cancer, first-line therapy, peritoneal metastasis, progression-free survival

## Abstract

**Objectives:**

To evaluate whether the modified intraperitoneal plus intravenous chemotherapy regimen as a first-line therapy for advanced epithelial ovarian cancer (EOC) in China can be well-tolerated or confer any potential benefit on survival.

**Methods:**

We evaluated the outcomes of women with newly diagnosed advanced-stage III-IV EOC treated with optimal cytoreductive surgery (<1 cm) and subsequent intraperitoneal plus intravenous chemotherapy or intravenous chemotherapy from January 2005 to December 2017 at two Gynecologic Oncology Centers in China. Kaplan-Meier survival analysis and Cox regression multivariate analysis models were performed to determine the toxicities and survival outcomes.

**Results:**

A total of 463 patients with stage III-IV EOC were enrolled. According to the propensity scores (1:2), 85 patients who received intraperitoneal plus intravenous chemotherapy (group A) were matched to 170 patients who received intravenous chemotherapy (group B). The median follow-up time was 41 months (range 6–155 months). However, there was no statistically significant difference in the median progression-free survival (PFS) (20 vs. 22 months, *P* = 0.351) or 3-year overall survival (OS) rate (80 vs. 78%, *P* = 0.749) between the two groups. R0 primary cytoreductive surgery was the only factor related to PFS (*P* = 0.028) and OS (*P* = 0.005) by Cox regression analysis. The incidence of grade 3/4 adverse events did not significantly differ between the two groups.

**Conclusion:**

The efficacy of intraperitoneal chemotherapy mainly comes from the intraperitoneal drug dose intensity and cumulative dose. High-efficiency and low-toxicity intraperitoneal chemotherapy regimens still need to be found and validated.

## Introduction

Peritoneal metastasis is a common occurrence in advanced epithelial ovarian cancer (EOC) (FIGO stage III-IV) and is associated with a dismal prognosis in the absence of an aggressive therapeutic approach (1, 2). Intraperitoneal chemotherapy has been proposed as an alternative approach for these patients to improve tissue concentrations and to reduce systemic toxicity (3, 4). Although 3 randomized controlled phase III trials (RCT GOG 104, 114, 172) have demonstrated that cisplatin-based intraperitoneal (IP) chemotherapy was an effective management for patients with EOC who underwent primary optimal cytoreduction (1–4), intraperitoneal chemotherapy has not been widely used in the treatment of ovarian cancer in either the United States or Europe until now (5). Clinicians always hesitant to use intraperitoneal chemotherapy because of the higher toxicity, inconvenience and catheter complications reported in GOG RCT trials (6–10). More recently, the fourth RCT trial, GOG 252, failed to show a survival advantage associated with either cisplatin or carboplatin intraperitoneal chemotherapy over dose-dense paclitaxel and carboplatin intravenous chemotherapy, when bevacizumab was included in all branches of the study (11). Therefore, the role of intraperitoneal chemotherapy as first-line treatment for primary epithelial ovarian cancer remains an area of uncertainty, which is the optimal drug and dose and the real benefit of intraperitoneal chemotherapy alone, especially whether it is superior to the present standard paclitaxel plus carboplatin intravenous chemotherapy in terms of efficacy and tolerability.

In China, intraperitoneal chemotherapy has been adopted in clinical practice for patients with FIGO stage III-IV EOC since the 2006 NCI recommendation. However, there have been few publications on population-level uptake and the survival outcomes of those interventions. Whether this modified intraperitoneal plus intravenous chemotherapy regimens can be well-tolerated or confer any clinical benefit has not been well-studied. Here, we performed a retrospective analysis of patients with newly diagnosed FIGO stage III-IV EOC who separately accepted intraperitoneal plus intravenous chemotherapy or intravenous chemotherapy alone after primary optimally cytoreductive surgery at the two largest gynecological cancer centers in Zhejiang, China.

## Materials and Methods

### Patients and Setting

We retrospectively evaluated the outcomes and toxicities of patients diagnosed with FIGO stage III-IV EOC patients who received intraperitoneal plus intravenous chemotherapy or intravenous chemotherapy alone after primary cytoreductive surgery at Women's Hospital, School of Medicine, Zhejiang University and Zhejiang Cancer Hospital from 2005 to 2017.

Those patients with newly diagnosed EOC who had optimal primary surgery followed by at least three cycles of platinum-based adjuvant chemotherapy were included in the study. The criteria for optimal cytoreductive surgery included no residual lesion visible by the naked eye (R0) and a diameter of residual disease <1 cm (R1). Exclusion criteria were the following preexisting medical conditions: (i) >1 cm of intraperitoneal residual disease (RD) at the end of cytoreductive surgery; (ii) patients who received neoadjuvant chemotherapy or received bevacizumab; (iii) patients who received maintenance treatment (bevacizumab or Poly ADP-ribose Polymerase inhibitors, PARPi etc.); (iv) patients who received nonplatinum chemotherapy; (v) FIGO stage I-II EOC; and (vi) incomplete clinical and follow-up data or died within 90 days of surgery.

**Figure 1:**
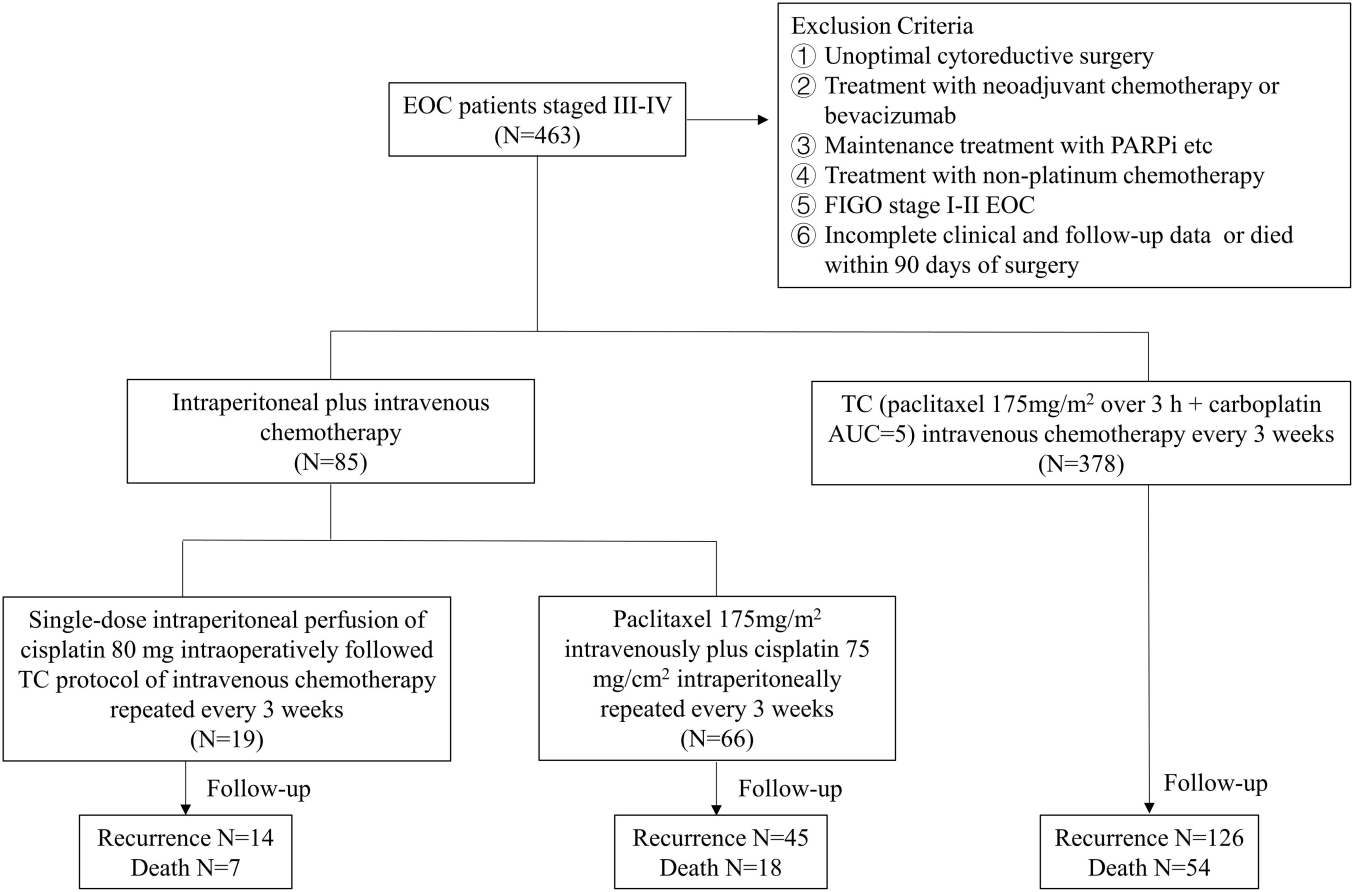
Flowchart of the study design.

Overall, the included patients were divided into two groups: intraperitoneal chemotherapy and no intraperitoneal chemotherapy. The intraperitoneal chemotherapy (A group) was further divided into two subgroups (A1 and A2 groups). Patients in the A1 group were treated with only a single dose (cisplatin 80 mg) of intraoperative intraperitoneal perfusion chemotherapy followed by at least 3 or more cycles of intravenous chemotherapy, and patients in the A2 group were treated with at least 3 or more cycles of intraperitoneal plus intravenous chemotherapy (paclitaxel 175 mg/m^2^ intravenously plus cisplatin 75 mg/m^2^ intraperitoneally repeated every 3 weeks). In single dose intraoperative intraperitoneal chemotherapy, cisplatin 80 mg was administered intraperitoneally in an open manner during the operation. The mutiple intraperitoneal chemotherapy with cisplatin (75 mg/m^2^) at a room temperature was administered over the course of 90 min by a closed technique through four intra-abdominal drains. The no intraperitoneal chemotherapy (B group) refers to patients who received 3 or more cycles of conventional intravenous chemotherapy (paclitaxel 175 mg/m^2^ over 3 h + carboplatin AUC = 5) without intra-abdominal medication. In each group, baseline patient data and disease and surgical characteristics were prospectively collected through the digital medical record system. The flowchart of the study design was shown in Figure 1.

All patients were followed up by telephone or outpatient visits up to September 1, 2020. The overall survival time (OS) was defined as survival months from the date of the primary cytoreductive surgery until death or the end of the observation period (Sep 1st, 2020), and patients alive after this date were censored. Progression-free survival (PFS) was described as the interval from the date of primary cytoreductive surgery to the date of recurrence or censored at the date of the last follow-up.

### Statistical Analysis

Differences in baseline data between the two groups were evaluated using a chi-square test. Propensity score matching (1:2) between groups A and B was performed to reduce the effect of selection bias. The median survival was calculated using the Kaplan-Meier method. The survival curves of OS and PFS were plotted with R-Studio for Windows. The 3-year survival rate was calculated by the life table method. Finally, the Cox regression univariate and multivariate analysis model was used to obtain hazard ratios (HRs) and their corresponding 95% confidence intervals (CIs). Additional statistical analysis was performed using SPSS software version 21.0, and bilateral *p* < 0.05 were considered statistically significant.

## Results

### Patient Characteristics and Treatments

During the 12-year study period, a total of 463 patients with stage III-IV EOC were eligible for analysis, including 85 received intraperitoneal chemotherapy and 378 received no intraperitoneal chemotherapy.

The demographics and clinical characteristics of these patients are outlined in Table 1. The median age was 53.0 years, and the range was 31 to 80 years. Approximately 87.5% (405/463) of patients had stage III disease, including 68% with stage IIIC disease, and 12.5% of patients had stage IV disease. Approximately 90.5% of patients had serous histology, 3.9% had endometrioid adenocarcinoma, and 5.6% had mixed ovarian cancers. Of the 463 patients, 213 (46.0%) underwent suboptimal R1 primary cytoreductive surgery, and 250 (54.0%) underwent R0 primary cytoreductive surgery. Before starting treatment, the median serum concentration of CA125 was 1106.3 U/ml (range, 13.9–6000.0 U/ml).

**Table 1:** Patients' characteristics of A and B groups before and after propensity score matching.

**Characteristic**	**A**	**B**	**Total**	***P***
**Before matching**				
Patients (*n*, %)	85 (18.4%)	378 (81.6%)	463	—
Age (median, range)	51 (34–70)	53 (31–80)	53 (31–80)	0.110
**Age group (*n*, %)**				
<50 years	32 (37.6%)	133 (35.2%)	165 (35.6%)	0.707
≥50 years	53 (62.4%)	245 (64.8%)	298 (64.4%)	
Serum CA125 level (U/ml)^*^				
Median (Range)	1244.3 (13.9–>10000.0)	1061.0 (14.8–>10000.0)	1106.3 (13.9–>10000.0)	0.106
<1000	35 (41.7%)	176 (46.9%)	211 (46.0%)	0.399
≥1000	49 (58.3%)	199 (53.1%)	248 (54.0%)	
**Individual cancer history (*n*, %)**				
Yes	3 (3.5%)	10 (2.6%)	13 (2.8%)	0.714
No	82 (96.5%)	368 (97.4%)	450 (97.2%)	
**Family history of cancer (*n*, %)**				
Yes	27 (31.8%)	96 (25.4%)	123 (26.6%)	0.277
No	58 (68.2%)	282 (74.6%)	340 (73.4%)	
**FIGO stage (*n*, %)**				
III	77 (90.6%)	328 (86.8%)	405 (87.5%)	0.468
IV	8 (9.4%)	50 (13.2%)	58 (12.5%)	
**Pathological classification (*n*, %)**				
Serous carcinoma	76 (89.4%)	343 (90.7%)	419 (90.5%)	0.540
Endometrioid adenocarcinoma	5 (5.9%)	13 (3.4%)	18 (3.9%)	
Mixed carcinoma	4 (4.7%)	22 (5.8%)	26 (5.6%)	
**Residual of surgery (*n*, %)**				
R0	26 (30.6%)	224 (59.3%)	250 (54.0%)	<0.001
R1	59 (69.4%)	154 (40.7%)	213 (46.0%)	
**No. of cycles completed (*n*, %)**				
3	1 (1.2%)	4 (1.1%)	5 (1.1%)	0.509
4	2 (2.4%)	11 (2.9%)	13 (2.8%)	
5	1 (1.2%)	18 (4.7%)	19 (4.1%)	
≥6	80 (95.2%)	346 (91.3%)	426 (92.0%)	
Median PFS (months)	20 (6–155)	25 (5–115)	24 (5–155)	0.457
3-year OS rate (95%CI)	80% (70.2%–89.8%)	79% (75.1%–82.9%)	79% (75.1%–82.9%)	0.849
**After matching**				
Patients (*n*, %)	85 (33.3%)	170 (66.7%)	255	—
Age (median, range)	51 (34–70)	52 (35–80)	52 (34–80)	0.842
**Age group (*n*, %)**				
<50 years	32 (37.6%)	70 (41.2%)	102 (40.0%)	0.684
≥50 years	53 (62.4%)	100 (58.8%)	153 (60.0%)	
Serum CA125 level (U/ml)^*^				
Median (Range)	1194.1 (13.9–7347.0)	1274.0 (14.8–9386.4)	1244.3 (13.9–9386.4)	0.881
<1000	35 (41.7%)	69 (40.8%)	104 (41.1%)	1.000
≥1000	49 (58.3%)	100 (59.2%)	149 (58.9%)	
**Individual cancer history (*n*, %)**				
Yes	3 (3.5%)	2 (1.2%)	5 (2.0%)	0.337
No	82 (96.5%)	168 (98.8%)	250 (98.0%)	
**Family history of cacer (*n*, %)**				
Yes	27 (31.8%)	49 (28.8%)	76 (29.8%)	0.664
No	58 (68.2%)	121 (71.2%)	179 (70.2%)	
**FIGO stage (*n*, %)**				
III	77 (90.6%)	143 (84.1%)	220 (86.3%)	0.180
IV	8 (9.4%)	27 (15.9%)	35 (13.7%)	
**Pathological classification (*n*, %)**				
Serous carcinoma	76 (89.4%)	155 (91.2%)	231 (90.6%)	0.314
Endometrioid adenocarcinoma	5 (5.9%)	4 (2.4%)	9 (3.5%)	
Mixed carcinoma	4 (4.7%)	11 (6.5%)	15 (5.9%)	
**Residual of surgery (*n*, %)**				
R0	26 (30.6%)	51 (30.0%)	77 (30.2%)	1.000
R1	59 (69.4%)	119 (70.0%)	178 (69.8%)	
**No. of cycles completed (*n*, %)**				
3	1 (1.2%)	2 (1.2%)	3 (1.2%)	0.385
4	2 (2.4%)	4 (2.4%)	6 (2.4%)	
5	1 (1.2%)	10 (5.9%)	11 (4.3%)	
≥6	80 (95.2%)	154 (90.6%)	235 (92.2%)	
Median PFS (months)	20 (6–155)	22 (6–115)	21 (6–155)	0.351
3-year OS rate (95%CI)	80% (70.2%–89.8%)	78% (70.2%–85.8%)	78% (72.1%–83.9%)	0.749

* *Not all patients had serum CA 125 level tested; the number here denotes the number of patients who had available data*.

The intraperitoneal chemotherapy (A group) and no intraperitoneal chemotherapy (B group) patients were well-balanced in relation to age, pathohistological tumor type, FIGO stage and other clinical parameters, except for the primary cytoreductive surgery status. The proportion of patients with R0 resection (59.3%) in the B group was significantly higher than that in the A group (30.6%) (*P* < 0.001). Furthermore, propensity score matching was used to compensate for the differences in clinicopathological parameters, including age, primary cytoreductive surgery status, cycles of chemotherapy and pathology. Based on the propensity scores, 85 patients who underwent intraperitoneal chemotherapy were matched to 170 patients who underwent no intraperitoneal chemotherapy (Table 1).

### Prognosis

**Figure 2:**
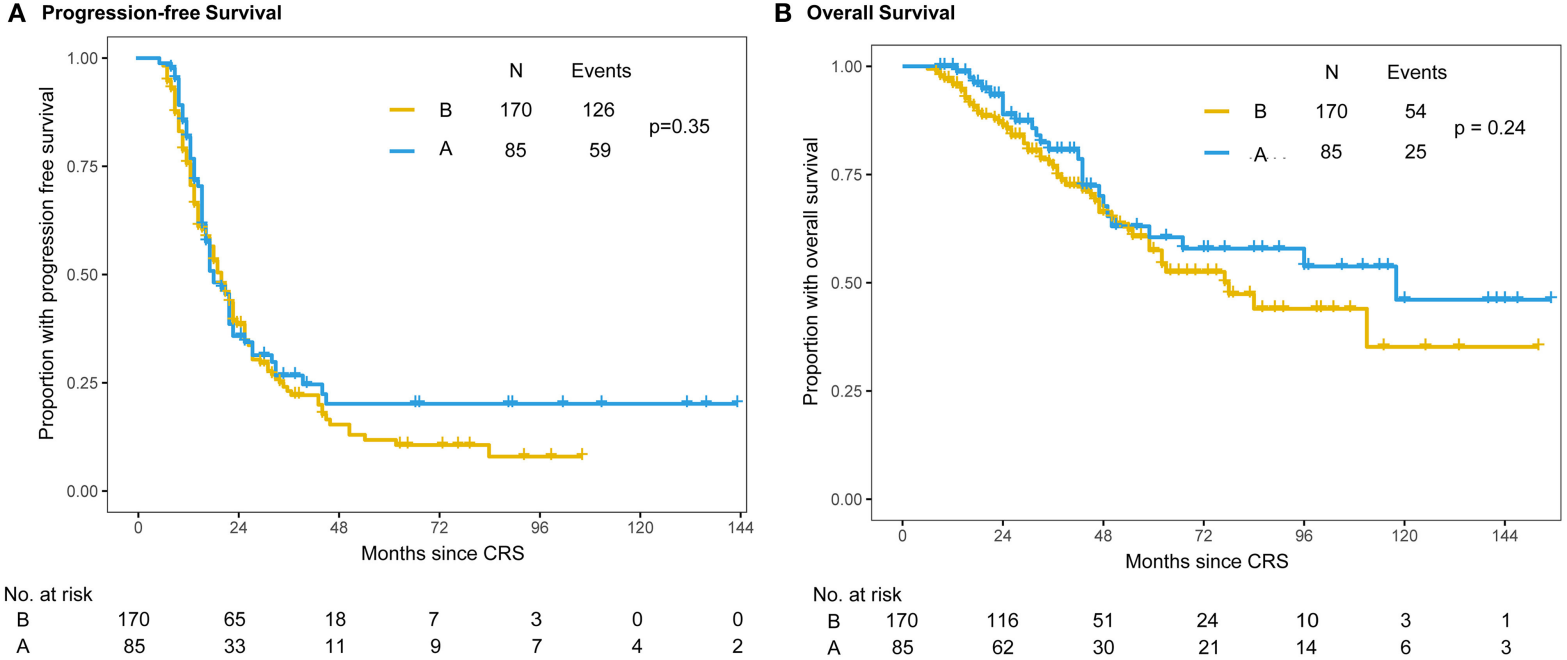
Kaplan–Meier distribution of progression-free survival time and overall survival time in propensity score-matched patients. Patients in the intraperitoneal plus intravenous chemotherapy (A group) and intravenous chemotherapy (B group) groups had no significant differences in PFS (*P* = 0.351) **(A)** or OS (*P* = 0.24) **(B)**.

At the time of the final follow-up (Sep 1st, 2020), the median follow-up was 55 months for the propensity score-matched patients with censored data. The median follow-up times were 48 months (range = 9−147 months) in the intraperitoneal chemotherapy (A group) and 41 months (range = 6−111 months) in the no intraperitoneal chemotherapy (B group). Of the 255 patients, 178 (69.8%) had progressed, 75 (29.4%) had died. The median PFS intervals were 20 months (range = 6−147 months) in the A group and 21 months (range = 6−107 months) in the B group, respectively (Figure 2). The 3-year OS was 80% (95% CI: 70.2%-89.8%) for the Group A and 73% (95% CI: 65.2%−80.8%) for the Group B respectively (Figure 2). In addition, there were no statistically differences both in PFS (*P* = 0.351) and 3-year OS (*P* = 0.24) between the two groups.

**Figure 3:**
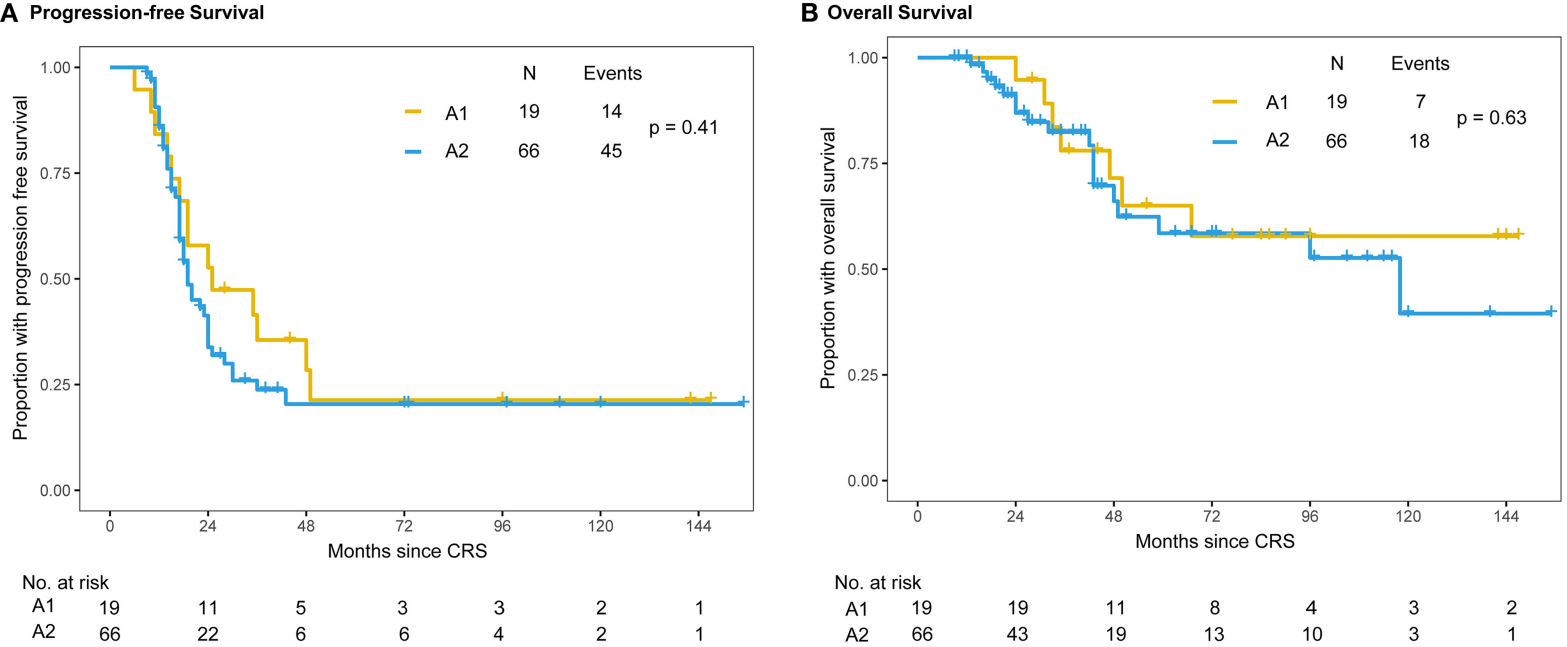
Kaplan–Meier distribution of progression-free survival and overall survival times in the subgroup of patients with intraperitoneal chemotherapy. Patients receiving single-dose intraperitoneal chemotherapy (A1 group) and multiple intraperitoneal chemotherapy (A2 group) had no significant differences in PFS (*P* = 0.41) **(A)** or OS (*P* = 0.63) **(B)**.

Within the A group, 19 patients belonged to A1 group and 65 patients belonged to A2 group. The median PFS intervals were 25 months in the A1 group and 19 months in the A2 group (Figure 3). There were no statistically significant differences in PFS (*P* = 0.41) or OS (*P* = 0.63) between the A1 and A2 chemotherapy subgroups. Until the censored date of our study, the 3-year survival rates were 78% (95% CI: 58.4%–97.6%) and 81% (95% CI: 69.2%–92.8%) in the A1 and A2 groups, respectively (*P* = 0.726) (Figure 3).

To further explore the potential variables relevant to the prognosis in the 255 propensity score matched patients, univariate and multivariate Cox regression analyses were applied. As showed in Table 2 that optimal R0 primary cytoreductive surgery was the only factor related to PFS (*P* = 0.028) and OS (*P* = 0.005).

**Table 2:** Univariate and multivariate analysis of progression free survival and overall survival in propensity score matched patients (*n* = 255).

		**Univariate analysis**			**Multivariate analysis**		
	**N, %**	**HR**	**95%CI**	***P***	**HR**	**95%CI**	***P***
**PFS**							
**Age group**							
<50 years	102 (40.0%)						
≥50 years	153 (60.0%)	1.097	0.817–1.473	0.536			
**FIGO stage**							
III	220 (86.3%)						
IV	35 (13.7%)	0.925	0.602–1.421	0.721			
**Residual in surgery**							
R0	77 (30.2%)						
R1	178 (69.8%)	1.437	1.040–1.985	0.028	1.437	1.040–1.985	0.028
Serum CA125 level (U/ml)^*^							
<1000	104 (41.1%)						
≥1000	149 (58.9%)	0.892	0.769–1.036	0.134			
**Treatment**							
Group A1	19 (7.5%)	1.000	—	0.474			
Group A2	66 (25.8%)	1.301	0.713–2.375	0.392			
Group B	170 (66.7%)	1.403	0.806–2.442	0.231			
**OS**							
**Age group**							
<50 years	102 (40.0%)						
≥50 years	153 (60.0%)	1.053	0.673–1.649	0.82			
**FIGO stage**							
III	220 (86.3%)						
IV	35 (13.7%)	0.508	0.205–1.258	0.143			
**Residual in surgery**							
R0	77 (30.2%)						
R1	178 (69.8%)	2.280	1.279–4.063	0.005	2.280	1.279–4.063	0.005
Serum CA125 level (U/ml)^*^							
<1000	104 (41.1%)						
≥1000	149 (58.9%)	0.936	0.747–1.172	0.563			
**Treatment**							
Subgroup A1	19 (7.5%)	1.000	—	0.475			
Subgroup A2	66 (25.8%)	1.213	0.505–2.910	0.666			
Group B	170 (66.7%)	1.519	0.687–3.356	0.302			

* *Not all patients had serum CA 125 level tested; the number here denotes the number of patients who had available data*.

### Toxicity

In this study, 92.0% (426/463) of patients received at least 6 cycles of chemotherapy. Generally, the level of toxicity was similar in all groups of patients. In the A1 group, all the 19 patients completed single-dose intraperitoneal perfusion chemotherapy intraoperatively and 6–8 cycles of intravenous chemotherapy. In the A2 group, 66 patients received an average of 5.6 intraperitoneal chemotherapy cycles, 93.9% (62/66) of patients completed all planned 6–8 cycles of intraperitoneal plus intravenous chemotherapy, and 4 patients changed to classical 3-weekly TC intravenous chemotherapy (2 patients due to intestinal obstruction and 2 patients due to catheter complications). In the B group, 378 patients received an average of 6.7 cycles of 3-weekly TC intravenous chemotherapy, 345 patients completed all planned 6–8 cycles of intravenous chemotherapy, and 33 patients did not complete all planned cycles (14 patients due to hematological toxicity, 2 patients due to chemoresistance and 17 patients due to personal reasons). The incidence rates of neutropenia and grade 3 or 4 thrombocytopenia did not significantly differ between the A and B groups.

## Discussion

GOG's three RCT studies have shown that first-line intraperitoneal combined with intravenous chemotherapy can significantly prolong the survival time of patients with stage III ovarian cancer after satisfactory tumor cytoreductive surgery (the overall risk of death is reduced by 20–30%) (2–4). The NCI Clinical Announcement (2006) and NCCN guidelines (2006) both recommend intraperitoneal chemotherapy or intraperitoneal plus intravenous chemotherapy as first-line chemotherapy for stage II-III ovarian cancer after satisfactory tumor cytoreductive surgery (residual lesion <1 cm), especially for stage III ovarian cancer with R0 reduction (5). However, up to now, intraperitoneal chemotherapy is still rarely used as the standard first-line treatment in clinical practice because of its high toxicity, inconvenience, risk of catheter-related complications and lack of a widely accepted optimal regimen. Currently, the most relevant criticism of pivotal GOG RCT intraperitoneal studies is the inequality of dose intensity between the treatment arms. In recent years, several gynecologic cancer centers have modified the regimen that originated from GOG 172 and tried to minimize the side effects, improve the convenience, and maintain the efficacy of intraperitoneal chemotherapy. Dash et al. (12) demonstrated that a modified dose reduction of day 2 cisplatin (75 vs 100 mg/m^2^) could reduce all recorded toxicities, and 63% of patients received at least 5 cycles compared with 51% in the GOG 172. However, they were not sure whether the reduction in cisplatin dose could maintain efficacy. A modified treatment protocol with 175 mg/m^2^ paclitaxel over 3 h on day 1 in the transvenous pathway and 75 mg/m^2^ cisplatin intraperitoneal pathway on day 2 every 3 weeks was the most popularly used intraperitoneal chemotherapy protocol for advanced epithelial ovarian cancer in China. Our findings indicated that compared with the standard 3-weekly TC regimen, this modified intraperitoneal plus intravenous chemotherapy regimen did not show advantages in PFS (*P* = 0.351) and OS (*P* = 0.24) between the A and B groups, although the complications of intraperitoneal chemotherapy were markedly reduced compared with GOG 172. The results of GOG 252 also showed that reducing cisplatin from 100 to 75 mg/m^2^ in intraperitoneal chemotherapy showed a significant decrease in toxicity, but compared with TC intravenous chemotherapy, neither intraperitoneal chemotherapy with cisplatin nor carboplatin showed a survival benefit (11). The use of bevacizumab has been cited as one of the reasons that there no longer appears to be an advantage to intraperitoneal chemotherapy in GOG 252 (11). However, intraperitoneal chemotherapy was not found to be superior to intravenous chemotherapy in this study, although we ruled out patients who received chemotherapy with bevacizumab or bevacizumab maintenance. Eoh et al. (13) reported that intraperitoneal carboplatin chemotherapy (AUC = 5) on day 1, intravenous paclitaxel 175 mg/m^2^ chemotherapy on day 2, intraperitoneal paclitaxel 60 mg/m^2^ chemotherapy on day 8, intravenous paclitaxel 135 mg/m^2^ chemotherapy on day 1, intraperitoneal cisplatin 100 mg/m^2^ chemotherapy on day 2, and intraperitoneal paclitaxel 60 mg/m^2^ chemotherapy on day 8 did not show an advantage in long-term survival benefits among advanced EOC patients when compared with standard 3-weekly TC intravenous chemotherapy. Therefore, we believe that the efficacy of intraperitoneal chemotherapy mainly comes from the higher intraperitoneal drug dose intensity and cumulative dose compared with the standard intravenous regimen, but this was based on the premise of sacrificing chemotherapy side effects.

Hyperthermic intraperitoneal perfusion chemotherapy (HIPEC), as another localized regional treatment strategy for advanced ovarian cancer, has been shown to have an improved prognosis when treated during surgery after gross tumor resection. Recently, the addition of hyperthermia has been considered to improve the efficacy of intraperitoneal chemotherapy and enhance the cytotoxic efficacy. Many trials have also confirmed the positive role of hyperthermia in the control of advanced ovarian cancer (14–16). However, HIPEC is highly expensive, lengthens the operation time by at least 1–2 h, and requires additional equipment, such as heaters and pumps. In addition, the HIPEC (drug, timing, duration, temperature) regimens differed among investigations. In China, most of the clinic centers did not have HIPEC equipment. The use of single-dose intraperitoneal chemotherapy at the time of surgery and/or in the immediate postoperative period is not only conducive to unified administration, but also can avoid some complications of long-term peritoneal access. Although single-dose intraperitoneal chemotherapy during surgery has been widely used, its clinical effectiveness is still unknown. Our study included 19 stage III-IV EOC patients who received a single dose of 80 mg unheated cisplatin during satisfactory tumor cytoreductive surgery and had no distinct effect on the prolongation of survival compared with the multiple intraperitoneal plus intravenous chemotherapy and the standard TC intravenous chemotherapy, although it was safe and feasible, with low toxicity. Yoon et al. (17) compared 26 patients who received only intravenous chemotherapy with the other 37 patients who received both intraperitoneal chemotherapy during surgery and adjuvant intravenous chemotherapy and found no distinct improvement in survival. In our pair of patients in the A1 group, a single cisplatin peritoneal infusion immediately after resection of the tumor did not affect the healing of the incision or the occurrence of anastomotic leakage in the gastrointestinal tract. The safety of intraoperative administration provides a basis for the further development of late abdominal hyperthermia. However, our results indicated that single-dose cisplatin intraperitoneal infusion immediately after tumor resection did not affect the healing of the incision or the occurrence of anastomotic leakage in the gastrointestinal tract and, to some extent, provided a basis for further development of late abdominal hyperthermia.

Recent studies demonstrated the advantage of intraperitoneal chemotherapy in recurrent EOC patients, including the primary cytoreductive surgery plus HIPEC and the secondary cytoreductive surgery followed HIPEC in platinum-sensitive recurrent EOC (18–21). According to the latest systematic review, the addition of HIPEC to cytoreductive surgery could significantly improve OS of both newly diagnosed EOC patients and recurrent EOC patients (18). But the subgroup analyzes indicated that patients who received a platinum-based HIPEC drug did not exhibit significantly improved OS (18), which is consistent with our results. Fagotti's study compared minimally invasive secondary cytoreduction plus HIPEC with open surgery plus HIPEC in isolated relapse from ovarian cancer and found that the minimally invasive approach for secondary cytoreductive surgery plus HIPEC is safe and efficient for single isolated relapse (21). In this study, we mainly focused on the front-line platinum-based chemotherapy regimens for patients with newly diagnosed advanced EOC, the effect of HIPEC on the outcome of patients with recurrent EOC will be studied in the future.

Previous reports have shown that patients with BRCA mutations may benefit the most from intraperitoneal chemotherapy. Unfortunately, in the cases in this study spanning 12 years, the mutational status of the BRCA genes was unknown in most of the patients. Therefore, the status of BRCA genes and the sensitivity of intraperitoneal chemotherapy could not be analyzed.

In conclusion, despite a growing number of evidences supporting the survival advantage to ovarian cancer patients treated with intraperitoneal plus intravenous chemotherapy, there are still many problems to be solved, such as what is the optimal regimen and dosage for Chinese population, what is the best timing of HIPEC and how to manage the side effects. The intravenous carboplatin plus paclitaxel regimen remains the clinical gold standard for the first-line treatment of advanced ovarian cancer. Based on the limitations of this retrospective study, more prospective studies are needed to confirm the findings.

## Data Availability Statement

The original contributions presented in the study are included in the article/supplementary material, further inquiries can be directed to the corresponding author/s.

## Author Contributions

YS, JX, and XX had the idea for, and designed the study. ST and YS were the coordinating principal investigator and analyzed and interpreted the results. YS wrote the first draft and was responsible for the overall planning and conduct of the study. JX revised the manuscript. ST and ZC enrolled patients and collected data. All authors were approved the final version of the manuscript.

## Funding

The research was supported by Natural Science Foundation of Zhejiang Province China (LBY21H160002).

## Conflict of Interest

The authors declare that the research was conducted in the absence of any commercial or financial relationships that could be construed as a potential conflict of interest.

## Publisher's Note

All claims expressed in this article are solely those of the authors and do not necessarily represent those of their affiliated organizations, or those of the publisher, the editors and the reviewers. Any product that may be evaluated in this article, or claim that may be made by its manufacturer, is not guaranteed or endorsed by the publisher.
